# Photocatalytic activity of Cr-doped TiO_2_ nanoparticles deposited on porous multicrystalline silicon films

**DOI:** 10.1186/1556-276X-9-543

**Published:** 2014-10-02

**Authors:** Anouar Hajjaji, Khaled Trabelsi, Atef Atyaoui, Mounir Gaidi, Latifa Bousselmi, Brahim Bessais, My Ali El Khakani

**Affiliations:** 1 Institut National de la Recherche Scientifique, INRS-Énergie, Matériaux et Télécommunications, 1650, Blvd. Lionel-Boulet, Varennes, QC J3X-1S2, Canada; 2 Laboratoire de Photovoltaïque, Centre de Recherches et des Technologies de l'Energie, Technopole de Borj-Cédria, BP 95, 2050 Hammam-Lif, Tunisia; 3 Laboratoire de Traitement et de Recyclage des Eaux, Centre de Recherches et des Technologies des Eaux, Technopôle de Borj Cedria, BP 273, 8020 Hammam-Lif, Tunisia; 4 Department of Applied Physics, University of Sharjah, P.O. Box 27272, Sharjah, United Arab Emirates

**Keywords:** Multicrystalline silicon, Porous silicon, Cr-TiO_2_, Photocatalysis, Amido black

## Abstract

This work deals with the deposition of Cr-doped TiO_2_ thin films on porous silicon (PS) prepared from electrochemical anodization of multicrystalline (mc-Si) Si wafers. The effect of Cr doping on the properties of the TiO_2_-Cr/PS/Si samples has been investigated by means of X-ray diffraction (XRD), atomic force microcopy (AFM), photoluminescence, lifetime, and laser beam-induced current (LBIC) measurements. The photocatalytic activity is carried out on TiO_2_-Cr/PS/Si samples. It was found that the TiO_2_-Cr/PS/mc-Si type structure degrades an organic pollutant (amido black) under ultraviolet (UV) light. A noticeable degradation of the pollutant is obtained for a Cr doping of 2 at. %. This result is discussed in light of LBIC and photoluminescence measurements.

## Background

In the past two decades, we observed a revival and a rapid expansion in titanium dioxide (TiO_2_) research activities. It was shown that this semiconductor has a strong photocatalytic activity compared to other semiconducting oxides, owing to its chemical stability and to the easy control of doping as well as electrical conductivity. This led to extensive works aiming to achieve TiO_2_-based nanoscale structures having high photocatalytic activities. The present work aims to present a novel photocatalytic structure consisting of Cr-doped TiO_2_ thin films deposited on multicrystalline porous silicon (PS). PS has been widely explored for its strong visible room-temperature photoluminescence (PL) and for its high potential application in photovoltaics (PV) [1,2]. TiO_2_ is often used in screen-printed solar cells [3,4] as a standard antireflective (AR) coating. PS-coated TiO_2_ was found to reduce the trapping of surface charge carriers and to enhance the PS photoluminescence stability and the optoelectronic properties of PS-based Si solar cells [5]. TiO_2_ films have been prepared by a variety of deposition techniques including chemical vapor deposition [6], sol-gel processing [7], reactive sputtering [8,9], and pulsed laser deposition (PLD) [10,11]. Previous investigations recognized TiO_2_ as a promising photocatalyst material for total destruction of common organic pollutants [12]. However, the effective photoexcitation of TiO_2_ requires irradiation in the ultraviolet (UV) region due to its large bandgap (3.2 eV), which leads to a merely 5% of solar energy absorption. Considerable efforts have been devoted to improve TiO_2_ photocatalytic performance in the visible light range. Such efforts include nitrogen, phosphate, and transition metal ion doping, and surface modification with dyes or quantum dots. The photocatalytic activity of TiO_2_ (mainly the anatase phase) doped with metallic ions showed that charge recombination processes largely depend on both type and doping level of the metallic element [13]. Small concentrations of transition metals (less than 1%) lead to a lot of traps for electrons and/or holes, which then increases the recombination processes [14]. However, this low concentration is not sufficient to increase visible light absorption in TiO_2_. On the contrary, a high doping level of about 5% to 10% may lead to a beneficial shift of the absorption edge toward visible light via a narrowing of the TiO_2_ bandgap; however, new recombination centers simultaneously take place, which then increases the recombination rates [15]. Therefore, it is important to solve the recombination problem by searching a compromise while moderately doping the TiO_2_ films. Cr doping was widely used to enhance light absorption of TiO_2_ in the visible light. However, it was observed that at a certain Cr doping level, an anatase-to-rutile structural transition [16] occurs, leading to a degradation of the photocatalytic activity of the material. In this work, Cr-doped TiO_2_ films were prepared using the co-sputtering deposition technique. Our aim consists of enhancing the visible light absorption efficiency of the TiO_2_ films via Cr doping. The effect of doping content on the photocatalytic efficiency of TiO_2_ has been studied by using the amido black dye.

PS has a large absorption spectrum lying from UV to near infrared, while TiO_2_ has an absorption limited to an energy radiation as high as 3.2 eV (anatase phase). The adjunction of TiO_2_ to PS could in principle generate further excitons that may enhance the photocatalytic activity, by injecting electrons (holes) in the conduction (valence) band of TiO_2_, which in turn enhance the photodegradation via an increase of the carrier lifetime.

## Methods

The experiments were carried out on p-type mc-Si wafer having a thickness of 330 μm and a resistivity of 0.5 to 2 Ω cm. PS is formed using the electrochemical anodization method, in an ethanoic hydrofluoric acid solution (HF (48 wt. %)/ethanol = 1:4). The current density and anodization time were set to 7 mA/cm^2^ and 15 min, respectively. Pure TiO_2_ and TiO_2_:Cr thin films were deposited by means of radio frequency (RF) magnetron co-sputtering (13.56 MHz) technique on the PS/mc-Si substrates. Technical details of the co-sputtering deposition conditions were reported elsewhere [16]. The TiO_2_ target was submitted to an RF power of 360 W, while the Cr element target was sputtered with variable power values (*P*_cr_) ranging from 8 to 150 W. The Cr content in the TiO_2_:Cr films increases with *P*_cr_ and can be adjusted, at will, from 2 to 17 at. % Cr [16]. The TiO_2_:Cr films were deposited at approximately 550°C, with a thickness of about 120 nm. The crystallographic structure was examined by means of the X-ray diffraction (XRD) technique using a Philips X'pert MPD X-ray diffractometer (Cu Kα radiation, PANalytical B.V., Almelo, The Netherlands). The surface morphology of the treated PS surfaces was investigated by atomic force microcopy (AFM). The PL properties were analyzed using a cooled GaInAs detector and Ar^+^ ion laser excitation source (*λ* = 488 nm). The effect of PS and TiO_2_/PS on the minority carrier diffusion length (*L*) of the active mc-Si material was evaluated by means of light beam-induced current (LBIC) measurements using the 632-nm wavelength of a He-Ne laser. The effective minority carrier lifetimes (*τ*_eff_) were measured by photoconductance technique (WCT-120). Finally, the photocatalytic activity of both undoped and Cr-doped TiO_2_ films was carried out on a special organic pollutant (amido black) using a multi-UV lamp photo-reactor having a UV output power of 1.4 W and a maximum spectral intensity at 352 nm. The photocatalytic activity was measured by measuring the concentration of amido black (AB) after 4 min of UV irradiation, using the 617-nm wavelength of a double beam UV-visible spectrophotometer.

## Results and discussions

**Figure 1:**
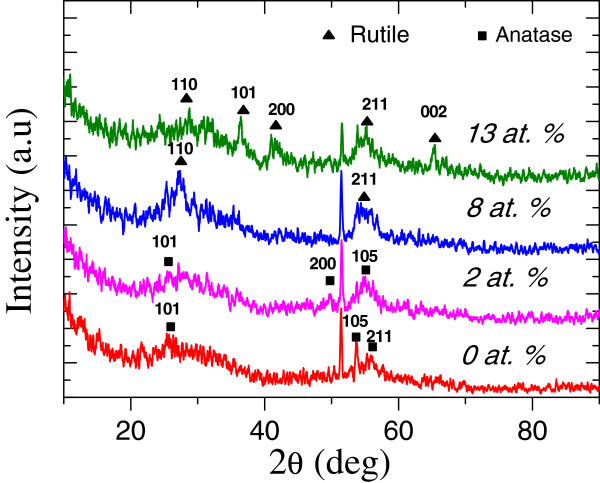
**XRD pattern of undoped and Cr-doped TiO**_**2**_**.**

XRD patterns (Figure 1) of undoped and Cr-doped TiO_2_ films, deposited on intrinsic silicon substrates at 550°C, are almost semi-crystalline. However, one may notice that an anatase-to-rutile phase transition occurs beyond a Cr doping concentration of 2 at. %.

**Figure 2:**
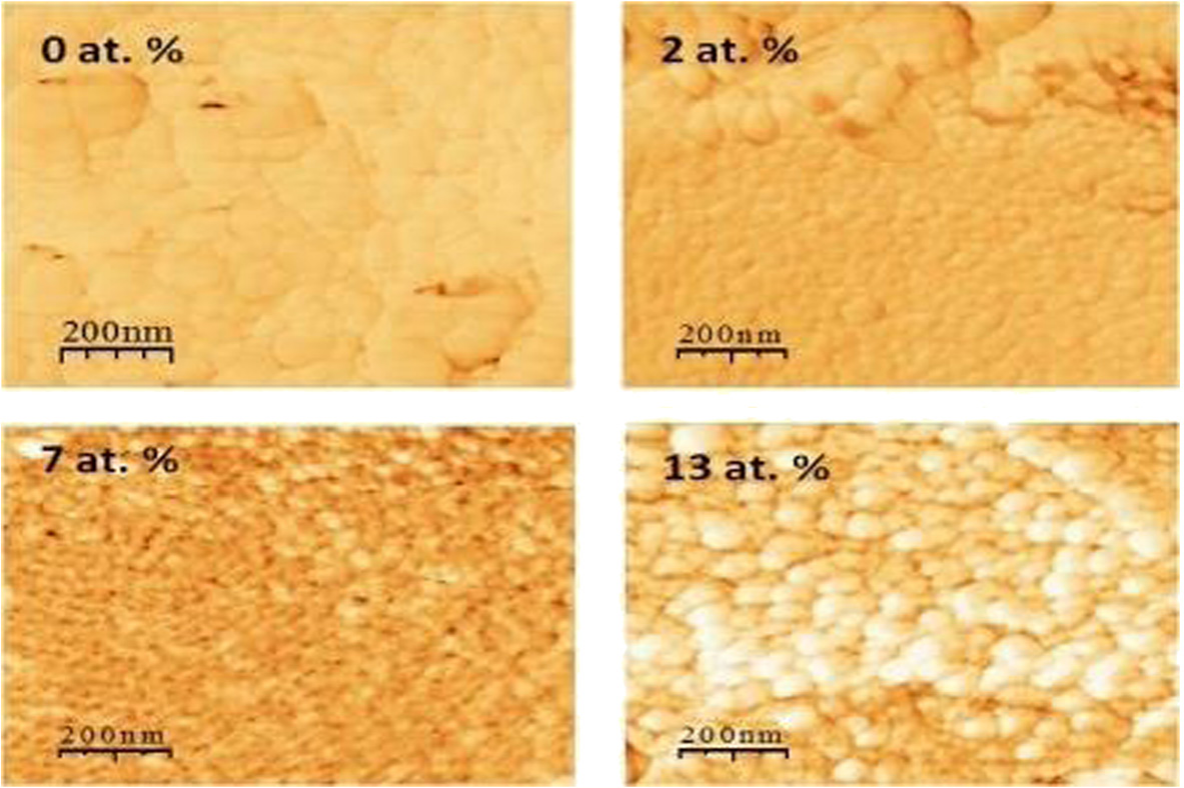
**AFM images of TiO**_**2**_**:Cr/PS/mc-Si for different Cr concentrations.**

Figure 2 shows surface AFM images of the TiO_2_:Cr/PS/mc-Si structure for different Cr concentrations. The structure of the Cr-doped films become further and further grainy (as compared to pure TiO_2_) starting from a Cr doping of 7 at. %.

AFM images show that incorporation of chromium induces a textural modification of the film (Figure 2). In a previous work, X-ray photoelectron spectroscopy [16] showed that the concentrations of CrO_2_ and Cr_2_O_3_ compounds increase with Cr content; it is obvious that for Cr concentration as high as 17 at. %, Cr exists in the TiO_2_ lattice as well in the form of CrO_2_ and Cr_2_O_3_ compounds. The level of oxidation state increases with decreasing size [16]. We also demonstrated that at high Cr content and mainly after annealing, Cr doping leads to anatase-to-rutile transition as well as to the formation of a small nanoparticle size. We also believe that a composite electrode made of TiO_2_ (or Cr-doped TiO_2_)/Cr_2_O_3_ forms, as has been already demonstrated by XPS [16].

LBIC measurement is commonly used to reach the effective diffusion length (*L*) of the minority carriers in mc-Si. The effective diffusion length *L* is calculated from the LBIC data by using the following expression [17,18]:

(1) $$I_{\text{LBIC}}\left(x\right)=A\;\exp\left(-\frac{x}{L}\right)x^{-n}$$

where *x* is the distance between the aluminum electrode [19] and the laser beam, *A* is a constant mainly depending on the intensity of the laser beam and the penetration depth [18], and *n* is a constant depending on the surface recombination velocity. A decrease of the factor *n* would be related to the reduction of the surface recombination velocity as a consequence of the passivating effect.

**Figure 3:**
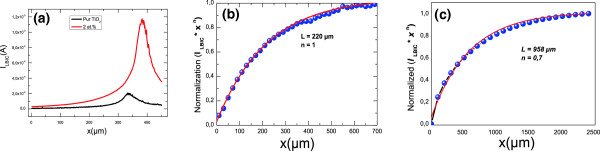
**Experimental and theoretical LBIC profiles. (a)** Experimental LBIC spectra, **(b)** adjustment of experimental and theoretical LBIC for TiO_2_/PS/mc-Si, and **(c)** TiO_2_:Cr (2 at. %)/PS/mc-Si.

LBIC analyses show a decrease of the *n* factor from 1 for the pure TiO_2_/PS/mc-Si to approximately 0.7 for TiO_2_:Cr (2 at. %)/PS/mc-Si. The effective diffusion length (Figure 3) was improved from 220 μm for the TiO_2_/PS/mc-Si to 958 μm for TiO_2_:Cr (2 at. %)/PS/mc-Si.

**Figure 4:**
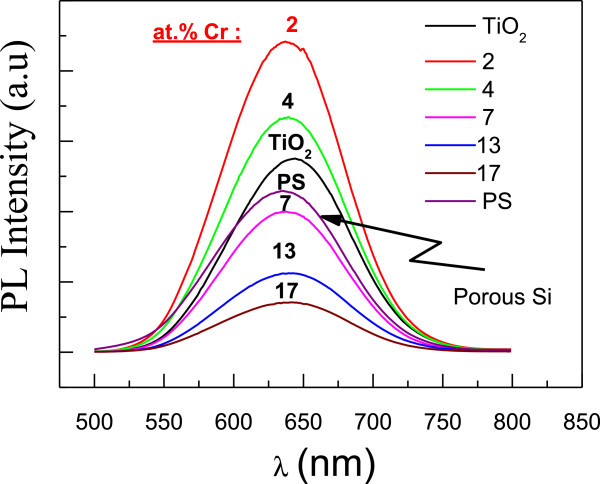
**PL spectra of PS coated with TiO**_**2**_**:Cr deposited at 550°C.**

Table 1 shows the evolution of the effective diffusion length (*L*) of the mc-Si with Cr concentration. We found that the greater *L* is associated to a low value of Cr concentration (2 at. %). This is consistent with PL measurements (Figure 4) where strong PL intensity was observed for a Cr doping concentration of 2 at. %. However, the increase in Cr concentration decreases the diffusion length; this can be explained by the great amount of the incorporated Cr atoms that may induce defects during the growth process [20]. Structural changes, especially textural modification together with appropriate Cr doping can notably enhance the photocatalytic property of the photocatalyst via a real change in the electrical conductivity of the doped films as shown in LBIC measurements (Figure 3a).

**Table 1:** **Evolution of the effective diffusion length of the mc-Si wafer after PS and TiO**_**2**_**:Cr/PS treatments**

	**Sample**				
	**mc-Si**	**PS/mc-Si**	**TiO**_**2**_**:Cr/PS/mc-Si**		
			**[Cr] = 0 at. %**	**[Cr] = 2 at. %**	**[Cr] = 13 at. %**
*L* (μm)	100	181	220	958	72

In the same way, the effective minority carriers lifetime (*τ*_eff_) is calculated using the following relation [21]:

(2) $$\frac{1}{\tau_{\text{eff}}}=\frac{1}{\tau_{\text{bulk}}}+\frac{2S}{W}$$

where *S* is the surface recombination velocity and *W* is the thickness of the mc-Si substrate. Table 2 presents the variation of the effective minority carrier lifetime of the mc-Si wafer after treatment with PS and TiO_2_:Cr/PS.

**Table 2:** **Evolution of the effective minority carrier lifetime of the mc-Si after treatment with PS and TiO**_**2**_**:Cr/PS**

	**Sample**				
	**mc-Si**	**PS/mc-Si**	**TiO**_**2**_**:Cr/PS/mc-Si**		
			**[Cr] = 0 at. %**	**[Cr] = 2 at. %**	**[Cr] = 13 at. %**
Lifetime (μs)	2	23	233	733	9

One may notice (Table 2) that PS or TiO_2_:Cr/PS-treated mc-Si samples have higher effective minority carrier lifetime values as compared to the untreated mc-Si wafer. First, the formation of PS improves the effective minority carrier lifetime of the mc-Si wafer from 2 to 23 μs; this value reaches a maximum of 733 μs after depositing a 2 at. % Cr-doped TiO_2_ film. Beyond this Cr concentration, the effective minority carrier lifetime decreases. The notable enhancement of the effective minority carrier lifetime for a Cr doping of 2 at. % (Table 2) would be due to a double step passivation; the first one is PS-based (reduction of the surface recombination velocity) [22], while the second one consists of a TiO_2_-based passivation of PS while choosing an adequate Cr doping. Figure 4 shows the PL spectra of TiO_2_:Cr/PS structures. We should keep in mind that the PL emission is exclusively coming from PS and that the most intense PL corresponds to a Cr concentration of 2 at. %; beyond this critical value, we notice a decrease of the PL intensity (Figure 4). Additionally, the PL band peak position is almost independent of Cr content. Usually, when PS is being oxidized, one may observe an enhancement of the PL intensity together with an energy shift of the PL band [23,24]. Therefore, one may presume that the decrease of the PL intensity for Cr doping higher than 2 at. % (Figure 4) is due to the change of the PS surface passivation, probably due to the formation of chromium oxides (textural modification) [16].

The decrease of the PL intensity (as Cr doping increases) could not be due to the anatase-to-rutile transition or to the doping itself, as only the PS surface is concerned; a surface textural (Figure 2) modification (due to an increase in Cr doping) could influence the PL intensity (increase of non-radiative charge carrier recombination in PS). On the other hand, the content of CrO_2_ and Cr_2_O_3_ forming during the co-sputtering of Ti and Cr together with the anatase-to-rutile transition depends essentially on Cr doping and experimental conditions [16].

**Figure 5:**
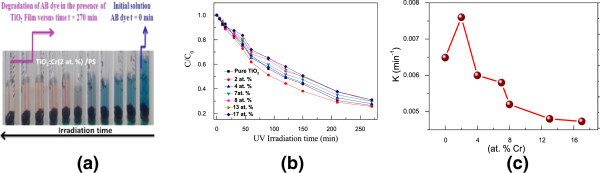
**AB discoloration, evolution of AB degradation, and variation of rate constant. (a)** Discoloration of amido black (AB) with TiO_2_:Cr/PS. **(b)** Evolution of the AB degradation as a function of UV light irradiation time for TiO_2_:Cr/PS films having different Cr concentrations; **(c)** Kinetic constant related to the discoloration of AB versus Cr concentration.

Figure 5a shows the discoloration of AB during its exposure to UV irradiation subsequent to photocatalysis in the presence of the TiO_2_:Cr/PS structure. Figure 5b depicts the discoloration kinetics of AB at different Cr concentrations. One may notice (Figure 5b) that the best photocatalytic degradation was obtained for a Cr doping of 2 at. %. It is worth noting a degradation of the photocatalytic response for Cr doping as high as 2 to 4 at. %. In fact, beyond this range of Cr concentration, an anatase-to-rutile phase transition occurs (Figure 1); knowing that anatase is the main active photocatalytic phase in TiO_2_, the gradual weakness of the photocatalytic activity of TiO_2_ (Figure 5b) would be due to the progressive disappearance of the anatase phase as Cr doping increases. It is well known that the photocatalytic degradation of the AB dye can be described by a first-order kinetic model [25], ln (Co/*C*) = *kt*, where Co is the initial concentration, *C* is the concentration at time *t*, and *k* is a constant.

Figure 5c shows the variation of rate constant *K* versus Cr concentration; *K* was calculated to be 0.0076 min^-1^ ± 2.165 10^-4^ for Cr (2 at. %)-doped TiO_2_. The photocatalytic activity of Cr(2 at. %)-doped TiO_2_ was found to be higher than that of the undoped TiO_2_ film; this can be related to the surface morphology and to the optical and optoelectronic properties of TiO_2_ doped with 2 at. % Cr. In fact, higher surface roughness (root-mean-square (RMS)) leads to an increase of the effective surface area and may then improve the photocatalytic activity; the RMS of the TiO_2_:Cr/PS structures increases from 17 to 43 nm as Cr doping varies from 0 to 4 at. %. Good photocatalytic response at an optimal concentration of 2 at. % Cr can also be explained by the increase in the minority carrier lifetime probably coming from carrier exchange with PS. This optimum could be explained by the fact that the presence of dopant prevents the recombination of exciton. Further increase in loading of Cr decreased the percentage of degradation of AB. This is due to the increased coverage of the semiconductor surface by the metal which decreases the surface adsorption sites expected to be available to the dye. The diminished penetration depth of light at higher Cr concentration could also prevent excitation of the semiconductor. In general, there are controversial results concerning the influence of Cr on the photocatalytic activity of the host material [26,27]. It is then difficult to compare the reported results due to the variety of synthesis and experiment conditions, which in turn have substantial impacts on the material bandgap and performance photoactivity [26-30].

The observed optimum Cr concentration of 2 at. % could be related to an optimum incorporation of Cr^3+^ and Cr^4+^ ions in the TiO_2_ matrix and probably to the formation of Cr nanoparticles (existence of Cr-Cr bonds [16]). The size of the nanoparticles can have a significant surface effect as the concentration of surface dangling bonds increases; the latter can serve as additional surface adsorption sites for the dye. On the other hand, oxidized small metallic nanoparticles are more active than reduced ones since the level of the oxidation state increases as the particle size decreases.

## Conclusions

It is found that TiO_2_ sputtered on multicrystalline porous silicon substrates can effectively enhance the photocatalytic activity of this well-known photocatalyst. A noticeable improvement of the photocatalytic activity of Cr-doped TiO_2_ was obtained at a critical Cr doping of 2 at. %, which in turn improves the effective diffusion length and the effective minority carrier lifetime of the silicon substrate.

Further works are in progress to analyze the effect of Cr doping on the photocatalytic activity of TiO_2_, especially when the material is deposited with other techniques on substrates other than multicrystalline silicon, since the elaboration procedure and even the method of doping can affect the properties of the catalyst.

## Competing interests

The authors declare that they have no competing interests.

## Authors' contributions

AH carried out the TiO_2_ elaboration and participated in the sample characterization. KT carried out the PS etching and participated in the sample analysis. AA participated in the photocatalysis measurement. MG participated in the elaboration, characterization process, and result interpretation, and is co-supervisor of the work. LB is responsible for the photocatalysis lab and participated in the measurement analysis. BB is co-supervisor of the work and participated in the sample analysis and result discussion. MAE is co-supervisor of the work and responsible for the sputtering deposition lab and participated in the sample analysis and result interpretation. All authors read and approved the final manuscript.
